# The Numerate Brain: Recent Findings and Theoretical Reviews on the Neurocognitive Foundations of Number Processing

**DOI:** 10.3389/fnhum.2012.00201

**Published:** 2012-07-05

**Authors:** Filip Van Opstal, Seppe Santens, Daniel Ansari

**Affiliations:** ^1^Ghent UniversityGhent, Belgium; ^2^University of Western OntarioLondon, ON, Canada

Numbers are omnipresent in our daily life. They are used to denote the date or time, the value of products, or to indicate the speed at which you drive your car. Indeed, numbers are part of our everyday life and as adults we use and manipulate them seemingly without any effort. But how do we do this? How is the meaning of numbers acquired and represented? And what brain mechanisms subserve the representation of number and mental operations involving numbers that guide our actions?

The burgeoning field of “Numerical Cognition” seeks to provide answers to these kinds of questions. Researchers in this field seek to understand the representation and the neural correlates of basic numerical processes and how these basic processes relate to higher-level mental operations of these foundational representations, such as mental arithmetic. It should be clear that the relation between the most elementary numerical skill, such as the extraction of the numerosity of a set of elements, and solving mathematical equations is quite complex and rather difficult to investigate. In fact, the many faces of numbers further complicate the matter. A number is a highly abstract symbol that can represent different things. It can symbolize a magnitude (cardinality; “4” could indicate the number of children you have) or the rank (ordinality; “4” could also refer to your youngest child when placed in chronological order). The same number can also be written in many forms: 4 is equal to IV, to “Four,” and to ••••. Furthermore, in mathematics, numbers can also be positive or negative, small or large, natural, or decimal numbers. Numerical cognition research aims at understanding how these different conceptions of numbers relate to each other and how we develop an understanding their meaning.

Over the last few decades these issues have been addressed by studying numerical skills in different animal species, human infants, and adults. However, the result of these years of focused research has not provided us with definite answers. Although some models of numerical cognition have dominated the research for many years, the basic representation of numbers remains debated until today. Even the mechanisms behind the most basic numerical skills, such as the comparison of two numbers, lack agreement in the research community. Indeed, the true nature of most of the commonly observed effects in simple numerical tasks, e.g., subitizing, the distance effect, the size congruity effect, or the SNARC-effect, remains obscure. On the other hand, the repeated observation of these effects in numerous studies indicates that they are genuine and that they could hold the key toward a proper understanding of number processing.

It appears that more agreement is reached on the brain areas related to basic number processing. Many brain imaging experiments in humans and monkeys, and studies on patients with brain lesions have shown a strong involvement of the horizontal segment of the intraparietal sulcus (hIPS) in number processing. However, despite the apparent agreement of a central role of the hIPS in number processing, the specificity of this area for numbers and the exact role of the hIPS in a more elaborate number processing brain network is still under investigation.

This Research Topic for *Frontiers in Human Neuroscience* covers a wide range of the remaining issues in numerical cognition. It discusses how numerical quantity is extracted from small sets of stimuli (Hyde, 2011), how the extraction of numerosity can be related to a system that extracts information from continuous dimensions (Henik et al., 2012) or to sensory-motor experiences (Ranzini et al., 2011). Furthermore, the causes of developmental dyscalculia are evaluated and the role played by symbolic (i.e., Arabic numerals) and non-symbolic (i.e., arrays of dots) in understanding this specific difficulty in mathematical processing is discussed (Noel and Rousselle, 2011). Research in the field of numerical cognition has grappled for to understand exactly how numerical and spatial processing are related. A number of contributions in this research topic shed further light on the association between number and space (Koten et al., 2011; Priftis et al., 2012; Van Dijck et al., 2012; Zorzi et al., 2012). Another question concerns the differences between automatic and intentional processing of number and the brain processes that mediate these different levels of number processing (Cohen Kadosh et al., 2012). Indeed, rather than confining number processing to a single area of the brain, a more elaborate network might be involved when a more elaborate number processing is needed, as in the processing of negative numbers (Blair et al., 2012), or doing mathematics (Grabner et al., 2011).
